# Biocompatible scaffolds based on collagen and oxidized dextran for endothelial cell survival and function in tissue engineering

**DOI:** 10.1002/elsc.202200140

**Published:** 2023-06-13

**Authors:** Fatemeh Sabet Sarvestani, Ali‐Mohammad Tamaddon, Ramin Yaghoobi, Bita Geramizadeh, Negar Azarpira

**Affiliations:** ^1^ Transplant Research Center Shiraz University of Medical Sciences Shiraz Iran; ^2^ Department of Pharmaceutical Nanotechnology and Center for Nanotechnology in Drug Delivery School of Pharmacy Shiraz University of Medical Sciences Shiraz Iran

**Keywords:** collagen, endothelial cells, laminin, oxidized dextran, scaffold

## Abstract

Angiogenesis is a vital step in tissue regeneration. Hence, the current study aimed to prepare oxidized dextran (Odex)/collagen (Col)‐hydrogels with laminin (LMN), as an angiogenic extracellular matrix (ECM) component, for promoting human umbilical vein endothelial cell (HUVEC) proliferation and function. Odex/Col scaffolds were constructed at various concentrations and temperatures. Using oscillatory rheometry, scanning electron microscopy (SEM), and cell viability testing, the scaffolds were characterized, and then HUVEC proliferation and function was compared with or without LMN. The gelation time could be modified by altering the Odex/Col mass ratio as well as the temperature. SEM showed that Odex/Col hydrogels had a more regular three‐dimensional (3D) porous structure than the Col hydrogels. Moreover, HUVECs grew faster in the Col scaffold (12 mg/mL), whereas the Odex (30 mg/mL)/Col (6 mg/mL) scaffold exhibited the lowest apoptosis index. Furthermore, the expression level of vascular endothelial growth factor (VEGF) mRNA in the group without LMN was higher than that with LMN, and the Odex (30 mg/mL)/Col (6 mg/mL) scaffold without LMN had the highest VEGF protein secretion, allowing the cells to survive and function effectively. Odex/Col scaffolds, with or without LMN, are proposed as a tissue engineering construct to improve HUVEC survival and function for angiogenesis.

**Abbreviations**
Col: collagen
3D: three‐dimensional
ECM: extracellular matrix
^1^H NMR: ^1^H nuclear magnetic resonance
HUVEC: human umbilical vein endothelial cell
LMN: laminin
Odex: oxidized dextran
TE: tissue engineering
VEGF: vascular endothelial growth factor

## 1 INTRODUCTION

Angiogenesis has been identified as the most crucial step in the regeneration and restoration of different tissues. New blood vessels formation seems to be vital to yield a successful cell transplantation rate in various tissue engineering (TE) constructs [1]. TE scaffold provides a three‐dimensional (3D) framework similar to the extracellular matrix (ECM) by using key elements such as biomaterials, seed cells, and biological active molecules for cells to attach and grow in vitro. The endothelial basement membrane consists of different ECM components such as laminin (LMN), collagen (Col) IV, and fibronectin that promote endothelial cell survival, growth, migration, and/or tube formation, and thus have proangiogenic properties [2, 3]. Therefore, selection of suitable biomaterials and construction of scaffolds mimicking the ECM microenvironment is an important strategy in TE [4, 5]. A favorable scaffold for promoting angiogenesis should be biocompatible and biodegradable and, more importantly, should exhibit a good interaction with endothelial cells. Among numerous biomaterials, Col‐based hydrogel scaffolds have been widely used in TE due to biocompatibility, ease of cross‐linking and functionalization, low antigenicity and inflammatory responses, biodegradability, and structural cell support [6, 7, 8, 9, 10, 11]. Native Col in solubilized form can be self‐assembled under physiological conditions into a hydrogel resembling loose native connective tissue for vascularization. According to Nguyen et al., Col scaffold was found to be a superior biomaterial for human mesenchymal stem cells and human umbilical vein endothelial cells (HUVECs) growth and simultaneous vascularization in the bone tissue [12]. However, its poor mechanical strength and fast degradation could pose limitations in TE applications [13]. Yet, these issues could be overcome by the combination of Col with other biomaterials such as dextran and adipic acid [14, 15, 16, 17].

Dextran is a polysaccharide, which is commonly used in biomedical fields. It has been used as a macromolecular prodrug and a plasma expander for several years [18]. It is available in a wide range of molecular weights and contains hydroxyl groups that make polymers highly hydrophilic and capable of chemical functionalization. Dextran is also biocompatible and can be degraded by dextranase in the liver, spleen, kidney, and colon [18, 19]. Polymeric cross‐linkers based on dextran are successfully prepared following a well‐known method that involves carbohydrate polymer oxidation by sodium periodate [20, 21]. The hydrophilic nature and reactivity of oxidized dextran (Odex) have been exploited in the synthesis of hydrogels as a crosslinker. The aldehyde groups of Odex may primarily react with Col amines without a need for any chemical reagent [22]. In the present study, a new approach was used to construct Col‐based hydrogels using Odex) as a cross‐linker with or without LMN. LMN, an ECM component with proangiogenic properties, was combined with Col to stimulate angiogenesis. LMN also binds to cell membranes via integrins and other plasma membrane components, which is essential for cell attachment and survival. Therefore, the study was aimed at developing a 3D construct of Odex/Col with or without LMN not only to improve the mechanical properties of the hydrogel, but also to maintain an acceptable HUVEC survival and growth condition for angiogenesis.

**PRACTICAL APPLICATION**

- Compared to the pristine Col hydrogel, the Odex/Col hydrogels had a more regular 3D microstructure with large pores.
- HUVECs grew faster in the pristine Col scaffold (12 mg/mL), whereas the Odex (30 mg/mL)/Col (6 mg/mL) scaffold had the lowest apoptosis index.
- HUVECs secreted the highest VEGF protein in the Odex (30 mg/mL)/Col (6 mg/mL) scaffold.

## 2 MATERIALS AND METHODS

### 2.1 Oxidized dextran preparation and characterization

Odex was prepared by oxidizing dextran using sodium (meta) periodate (NaIO_4_) to introduce aldehyde groups with a common recipe reported in the literature [23, 24]. Briefly, 1 g dextran (35,000–45,000 MW, sigma, Germany) and 190 mg NaIO_4_ (Merck, Germany) were dissolved in deionized water. Then, the mixture was stirred in the dark at room temperature for 24 h. The reaction was stopped by adding glycerol (60 μL) and the product was precipitated by adding NaCl (300 mg) and two‐fold excess of ethanol 99%. The obtained solution was dialyzed against deionized water for 3 days to remove unreacted NaIO_4_. The final Odex solution was freeze‐dried and stored at 4°C.

### 2.2 Fourier‐transform infrared spectroscopy

The dried powder of dextran and Odex were used for Fourier‐transform infrared spectroscopy (FTIR) analysis using a Thermo Scientific instrument (made in USA) in attenuated total reflectance (ATR) mode. All spectra were acquired in the 4000−400 cm^−1^ range with a resolution of 4 cm^−1^ for 20 scans. The data were collected, and the graphs were prepared using the Origin software.

### 2.3 ^1^H Nuclear magnetic resonance

^1^H nuclear magnetic resonance (^1^H NMR) spectroscopy was performed to characterize the Odex product in comparison with dextran. To that aim, 10 mg of each dried sample was dissolved in 1 mL of D_2_O. The solutions were then poured into NMR tubes and the spectra were acquired with a 500 MHz Fourier transform NMR spectrometer (Bruker, Germany) at room temperature. The spectra were collected and analyzed using the Mnova NMR software.

### 2.4 Determination of dextran oxidation degree

The aldehyde content of Odex was determined using the hydroxylamine hydrochloride method described by Zhao and Heindel [25]. Briefly, 0.1 g of the purified product was dissolved in 0.25 M NH_4_OH·HCl–methyl orange solution and was allowed to dissolve at room temperature for 2 h. The hydrochloride molecules released from the reaction product were quantified by titrating the polymer solution against a standard NaOH solution to reach the original color. The degree of oxidation was expressed as moles of aldehyde produced per mole of dextran monomers in the sample using the following equation:

$$\begin{array}{ccc} & & \mathrm{Oxidation}\left(\%\right)=\mathrm{Volume}{\left(\mathrm{mL}\right)}_{\mathrm{NaOH}}\times 10^{-3}\mathrm{moles}\\ & & \;\times \;M_{\mathrm{NaOH}}\times \mathrm{Molecular}\;\mathrm{weigh}t_{\mathrm{dextran}}/\;\left(\mathrm{Weight}{\left(g\right)}_{\mathrm{Odex}}\right)\\ & & \;\times \;100 \end{array}$$

### 2.5 Construction of scaffolds

To prepare Odex/Col hydrogel scaffolds, the Col solution (12 mg/mL, Sivan, Iran) was neutralized by adding 1 mol/L sodium hydroxide. Afterwards, the neutralized Col solution and the Odex solution (6%, prepared in culture media) were mixed in various mass ratios at 4°C (in 24 well plate, Corning, USA) and were allowed to react at 25 and 37°C [26]. The mixed Odex/Col and Col solutions were used for further experiments.

### 2.6 Scanning electron microscopy

The prepared Odex/Col hydrogels were washed in phosphate buffered saline (PBS, pH 7.4) and were fixed in 2.5% glutaraldehyde (Sigma, Germany) for 1 h. The hydrogels were dehydrated in serial dilutions of 25%, 50%, 75%, and 95% ethanol for 30 min. Finally, they were frozen at −80°C and were dried with freeze‐drier equipment for 48 h. The samples were coated with gold using a sputtering coater (Q150R‐ES, Quorum Technologies, England) and were evaluated using a scanning electron microscope (SEM) (TESCAN‐Vega 3, Czech Republic). The images were analyzed using the ImageJ software [27].

### 2.7 Rheology measurements

The rheological behaviors of the Odex/Col mixture and the Col solution were investigated in a strain‐controlled mode using a Physica MCR 302 rheometer (Anton Paar, Austria) with a cone‐and‐plate geometry of 1° incline and a 60 mm diameter (CP 60/1). To minimize evaporation, a solvent trap was employed, and low viscosity mineral oil was applied around the sample. To determine the effect of the incubation temperature on the rheological behavior of the pristine Col solution and the Odex/Col mixture, the storage moduli (*G*′) and loss moduli (*G*″) were monitored as a function of time at 1 Hz and 0.01 strain for 1 h at two different temperatures (25 and 37°C). A Peltier temperature control device was used to control the sample temperatures. All preparations were freshly made at the time of experiment.

### 2.8 3D cell culture

For a 3D culture, HUVECs (purchased from Pasteur Institute, Tehran, Iran) were cultured first in the DMEM‐F12 media (Gibco, Germany) supplemented with 10% FBS (Gibco, UK) and 1% penicillin–streptomycin (Pen/Strep, Sigma, Germany) with 5% CO_2_ at 37°C. The HUVECs were washed with PBS and were detached with trypsin at 37°C for 5 min when they were 70%−90% confluent. After cell counting, 50,000 cells were seeded into the Odex/Col 3D hydrogels that prepared in the aforementioned section and were incubated at 37°C for 72 h.

### 2.9 Cell viability assay

To determine cell proliferation and viability in the 3D scaffolds, Live–Dead fluorescence microscopy and Alamar blue assay were performed according to the previously published method [28, 29]. For Live–Dead fluorescence microscopy, 5 mg/mL fluorescein diacetate (FDA) and 2 mg/mL propidium iodide (PI) were used to detect the live and dead cells, respectively. The stained HUVECs were observed under the fluorescent microscope (CKX53, Olympus, Japan). For Alamar blue assay, the culture medium was aspirated after 72 h and the cell‐impregnated constructs were washed with sterile PBS (pH 7.4) and incubated with a freshly prepared Alamar blue reagent (G‐Biosciences, St. Louis, USA) in the complete culture medium (10% v/v) for 4 h. The fluorescence intensity was read by a fluorescence plate reader (BMG LABTECH, POLAR star Omega, German) at the excitation *λ*_max_ of 544 nm and emission *λ*_max_ of 590 nm. Matrigel was used as the gold standard [30].

### 2.10 Scaffold construction via LMN

Based on rheometry, SEM, and cell viability experiments, the most viable 3D hydrogel was chosen for constructing scaffolds with LMN. To that aim, the neutralized Col solutions were mixed with LMN (Sigma, Germany) at the final concentration of 50 μg/mL [31] and were added to the Odex solution (6%) at 4°C, as described before. After that, 50,000 cells were seeded into the 3D scaffold and were incubated at 37°C for 72 h. The cell proliferation was then compared to that of the scaffold without LMN.

### 2.11 Gene expression in HUVECs

Real‐time reverse transcription polymerase chain reaction (RT‐PCR) was used to compare the treated group (HUVECs embedded in the Odex/Col scaffolds or Matrigel) and the control group (HUVECs without scaffold, *n* = 3) regarding the expressions of BAX, BCL2, vascular endothelial growth factor (VEGF), and HIF‐1a genes. First, RNA was extracted using the RNA‐Sol isolation reagent (Alphabio, Canada). Then, cDNAs were synthesized using the Prime Script TM RT Reagent Kit (Takara, Japan). The primers were designed by the NCBI tool Primer BLAST, and *GAPDH* was considered the housekeeping gene. The primer pairs have been listed in Table 1. Gene amplification was evaluated by SYBR Premix Ex TaqTM II (Takara, Japan) using Applied Biosystems Step One Plus Real‐Time PCR system (ABI, USA) in 40 cycles. The fold changes of each gene were calculated by the Livac (2^−ΔΔCT^) method. Melt curves were also analyzed to confirm the specificity of the reaction at the end of the program.

**TABLE 1:** Primers used for determining the expressions of various genes by real‐time reverse transcription polymerase chain reaction (RT‐PCR).

Genes	Sequences		Length (bp)
*GAPDH*	Forward	GCTCATTTCCTGGTATGACAACG	127
	Reverse	CTCTCTTCCTCTTGTGCTCTTG	
*BAX*	Forward	TTCTGACGGCAACTTCAACT	134
	Reverse	GGAGGAAGTCCAATGTCCAG	
*Bcl2*	Forward	GATGGGATCGTTGCCTTATGC	105
	Reverse	CAGTCTACTTCCTCTGTGATGTTGT	
*VEGF‐A*	Forward	CTTCAAGCCATCCTGTGTGC	111
	Reverse	ATCCGCATAATCTGCATGGTG	
*HIF1a*	Forward	GCAGCAACGACACAGAAACT	131
	Reverse	TTCAGCGGTGGGTAATGGAG	

*Bax*, Bcl‐2‐associated X protein; *Bcl‐2*, B‐cell lymphoma 2; *GAPDH*, glyceraldehyde 3‐phosphate dehydrogenase; *HIF1ɑ*, hypoxia inducible factor 1 subunit alpha; *VEGF*, vascular endothelial growth factor.

### 2.12 VEGF protein secretion

The condition media of the HUVECs‐laden 3D scaffolds were collected after 72 h for VEGF secretion measurement using human VEGF ELISA kits (Life Technologies, France) according to the manufacturer's instructions. The results were presented as pg/mL.

### 2.13 Statistical analysis

All statistical analyses were performed using the SPSS 19.0 software (SPSS Inc., Chicago, USA). The results were expressed as mean ± SD. The data were analyzed using one‐way ANOVA followed by Tukey's post‐hoc test, and *p* < 0.05 was considered statistically significant.

## 3 RESULTS

### 3.1 Characterization of oxidized dextran (Odex)

The chemical structure of Odex was analyzed by FTIR and ^1^H NMR methods in comparison with dextran (Supplementary File 1 [S1]). In the FTIR spectra of both compounds, the broad peak at 3500 cm^−1^ corresponded to the OH stretching vibration of polysaccharides [32]. The band at 2931 cm^−1^ was similarly assigned to C–H stretching vibration [32, 33]. In addition, the sharp band at 1009 cm^−1^ and the shoulder at 1162 cm^−1^ could be related to the asymmetrical C–O vibrations [34]. Weak bands at 767, 849, and 915 cm^−1^ revealed the presence of α‐glucopyranose ((1→3)‐α‐d‐glucan, a ring structure of glucose molecules)in both spectra of dextran and Odex, indicating that the polymer backbone was preserved during the oxidation reaction [35]. Furthermore, the bands at 1647 and 1654 cm^−1^ in the Odex and dextran spectra were more likely related to O–H bending. However, the formation of aldehyde carbonyls (H–C = O) in Odex could not be ruled out, because the related band overlapped by the high intensity O–H bending band [36, 37]. The ^1^H NMR spectra of dextran and Odex have been depicted in Figure 2 S1. The spectrum revealed multiple peaks at 3.2−3.9 ppm, corresponding to the protons of the glucopyranosyl ring in dextran. The anomeric proton from the glucopyranose ring of dextran with α−1, six linkages was found at *δ* 4.9 ppm, while the peak at *δ* 5.3 ppm was attributed to the anomeric proton in dextran with α−1, three linkages [21, 38]. Aldehyde proton could not be detected due to the reversible formation of hemiacetal or hemialdal through the reaction of an aldehyde with the neighboring hydroxyl groups [23, 39]. To support the spectroscopic findings, the hydroxylamine hydrochloride method was used to confirm the oxidation degree of 46% for the prepared Odex.

**FIGURE 1:**
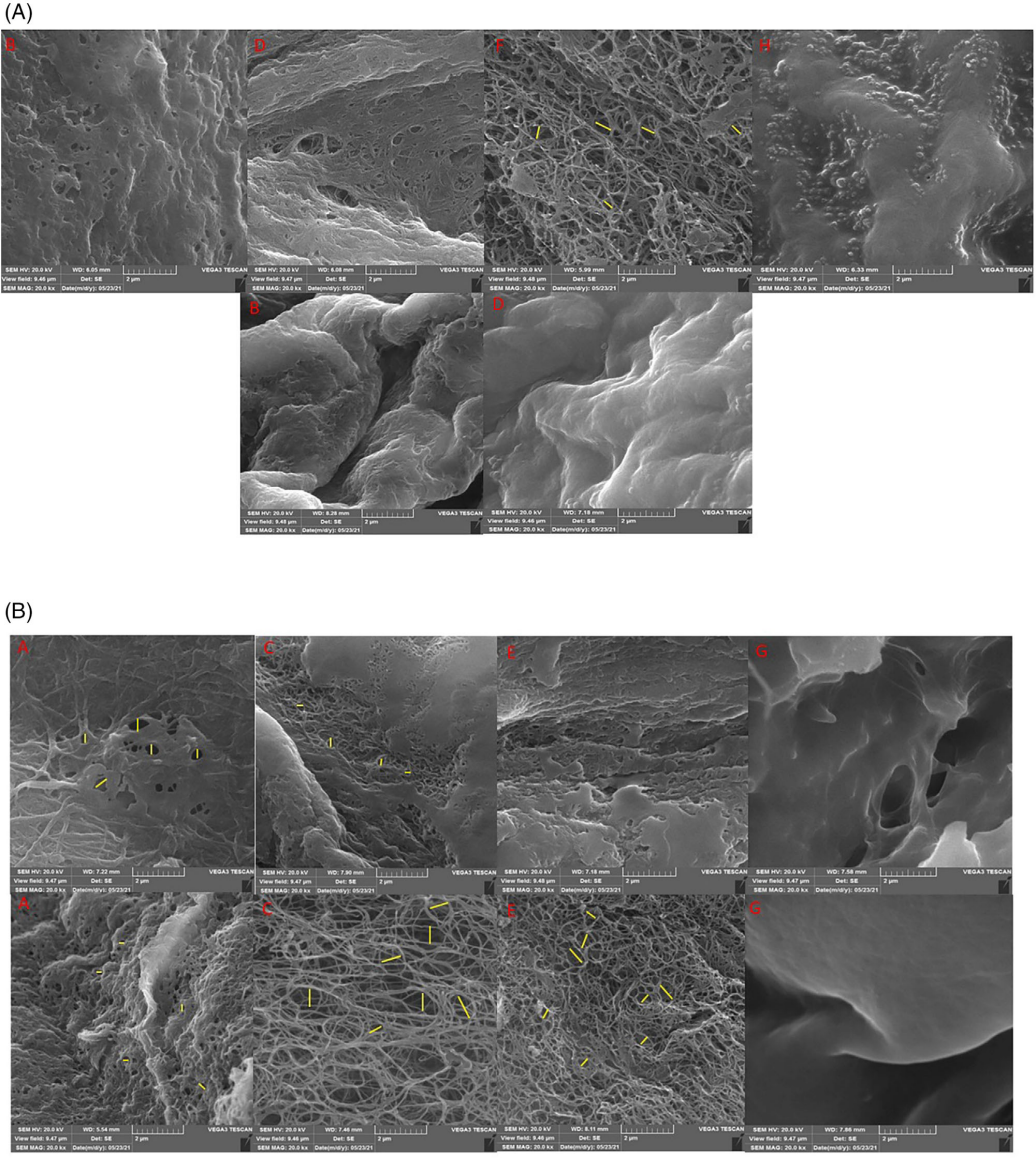
Scanning electron microscopy (SEM) images of hydrogel scaffolds with collagen (Col) and oxidized dextran (Odex). Upper and lower panels are related to the scaffolds prepared at 25 and 37°C, respectively. The scaffolds diameters and pores sizes were quantified by ImageJ software (referred to Table 2). A: Col (12 mg/mL); B: Col (6 mg/mL); C: Odex (30 mg/mL)/Col (6 mg/mL); D: Odex (30 mg/mL)/Col (3 mg/mL); E: Odex (40 mg/mL)/Col (4 mg/mL); F: Odex (40 mg/mL)/Col (2 mg/mL); G: Odex (45 mg/mL)/Col (3 mg/mL); H: Odex (45 mg/mL)/Col (1.5 mg/mL). Pore size distribution is indicated by yellow lines where applicable.

The SEM images of Odex/Col scaffolds and Col alone have been presented in Figure 1. Col alone resulted in larger pores at 25°C compared to 37°C. Nonetheless, large pores were formed at 37°C when Col was combined with Odex. Additionally, fibrous nanostructures with a lot of large pores were found in groups “C” and “E” at 37°C and in groups “D” and “F” at 25°C (Table 2). Col hydrogels had larger pores and fiber diameters at 25°C than at 37°C. Similarly, large pores were obtained if the high concentration of Odex was combined with the low Col concentration (group “C”) at 37°C (Table 2).

**TABLE 2:** Fiber diameters and pore sizes of the constructed hydrogel scaffolds.

Groups	Fiber diameter (μm)	Pore size (μm)
C‐37°C	0.08^a^ ± 0.01	0.48^a b^ ± 0.10
C‐25°C	0.05 ± 0.01	0.21 ± 0.11
E‐37°C	0.06^b^ ± 0.01	0.25^b^ ± 0.12
A‐37°C	<0.01^a b c^	0.16^a c^ ± 0.09
A‐25°C	0.18^c^ ± 0.03	0.39^c^ ± 0.06
F‐25°C	0.09 ± 0.02	0.42 ± 0.13

Groups with dissimilar superscripts were significantly different at *α* = 0.05 (*p* < 0.05). Col: collagen; Odex: oxidized dextran; A: Col (12 mg/mL); C: Odex (30 mg/mL)/Col (6 mg/mL); E: Odex (40 mg/mL)/Col (4 mg/mL); F: Odex (40 mg/mL)/Col (2 mg/mL). Col, collagen; Odex, oxidized dextran.

To investigate the hydrogelation of the Odex/Col mixtures, oscillatory rheology tests were used. The gelation time (min), elastic modulus (*G*′) after 30 min, and zero‐shear viscosity (Pa s) at 25 and 37°C have been summarized in Table 3 (for detailed information, please refer to Supplementary File 2). Gelation time was found to be related to Col concentration, Odex/Col ratio, and temperature. At both 25 and 37°C, hydrogel scaffolds formed quickly (<1 min) in groups “A,” “B,” “C,” and “E”. However, gelation occurred rapidly (<1 min) in group “D” only at 25°C. According to our findings, Col concentrations greater than 3 mg/mL resulted in the faster development of hydrogels. After a few minutes, the hydrogel scaffold in groups “F” and “H” with Col concentrations less than 3 mg/mL demonstrated a gel‐to‐sol transition (i.e., not stable over time). After a 30‐min incubation, *G*′ modulus reduced when the Odex/Col ratio was increased at 25°C. Importantly, unlike groups “A” and “B,” groups “C,” “E,” and “G” had substantially more stiff scaffolds at 37°C than at 25°C. Importantly, *G*′ modulus in group “C” at 37°C was roughly 10 times higher than pure Col (group “A”). Group “E” had the largest level of *G*′ modulus at 37°C after 30 min. The zero‐shear viscosity was also correlated well to the *G*′ values in all groups, except for group “A” which had a higher value compared to group “E”.

**TABLE 3:** Rheometry analysis of hydrogel scaffolds at 25 and 37°C.

Temp	Groups	Gelation/crossover time (min)	*G*′_30 min_ (Pa)	Zero‐shear viscosity (Pa s)	Groups	Gelation/crossover time (min)	*G*′_30 min_ (Pa)	Zero‐shear viscosity (Pa s)
25°C	A	<1, NA	650	83,053	B	<1, NA	140	19,730
	C	<1, NA	60	6080	D	<1, NA	60	1842
	E	<1, NA	50	4503	F	70	10	100
	G	1	15	1291	H	75	5	277
37°C	A	<1, NA	300	42,073	B	<1, NA	70	1693
	C	<1, NA	3500	1500	D	45	100	3656
	E	<1, NA	6200	45,000	F	Gel‐to‐sol transition, 32	60	1391
	G	11	5500	1836	H	Gel‐to‐sol transition, 15	100	1169

A: Col (12 mg/mL); B: Col (6 mg/mL); C: Odex (30 mg/mL)/Col (6 mg/mL); D: Odex (30 mg/mL)/Col (3 mg/mL); E: Odex (40 mg/mL)/Col (4 mg/mL); F: Odex (40 mg/mL)/Col (2 mg/mL); G: Odex (45 mg/mL)/Col (3 mg/mL); H: Odex (45 mg/mL)/Col (1.5 mg/mL). Col, collagen; NA, not available; Odex, oxidized dextran.

### 3.2 HUVECs viability and functionality

**FIGURE 2:**
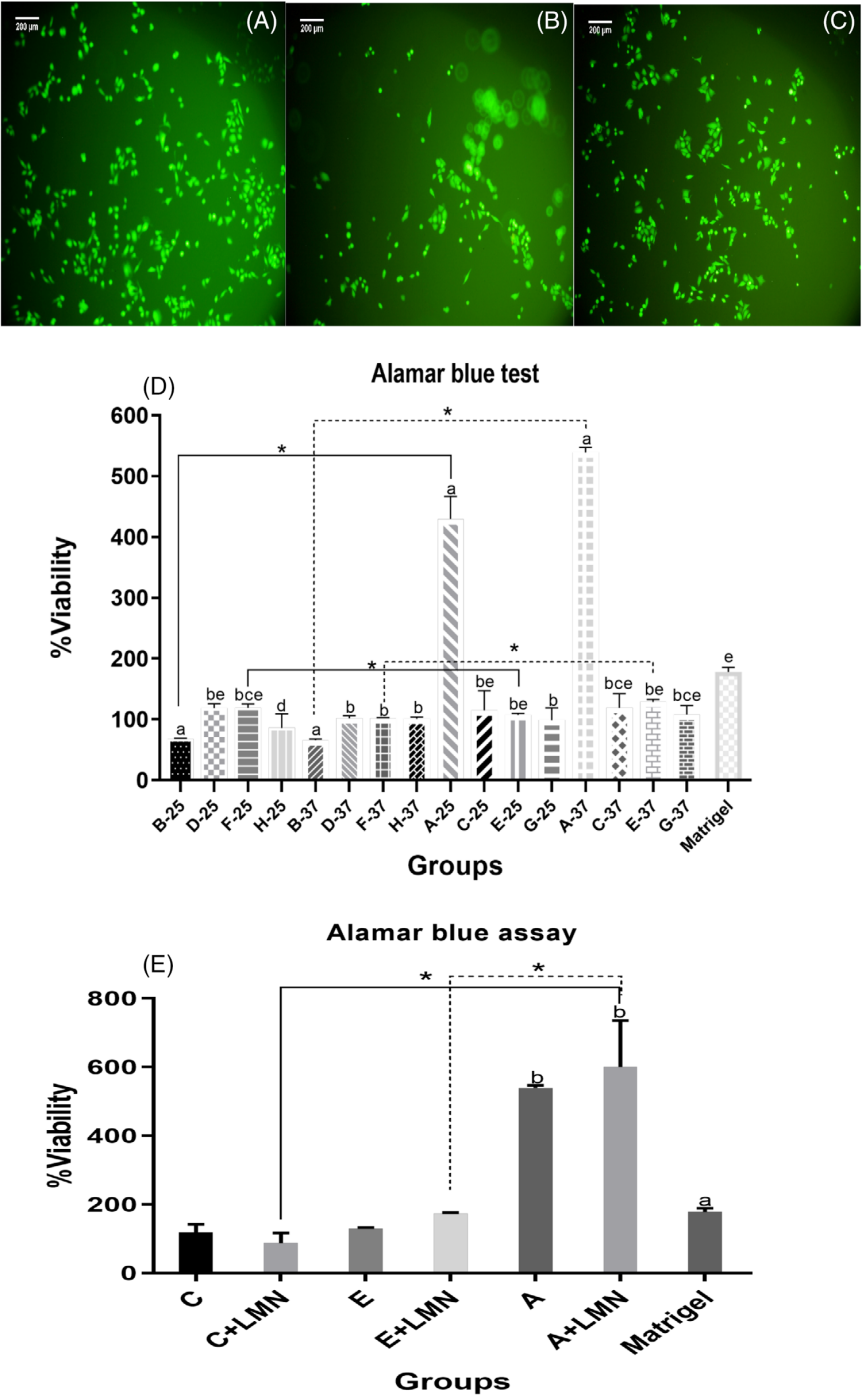
Fluorescein diacetate/propidium iodide and Alamar blue assays. Fluorescent images of human umbilical vein endothelial cells (HUVECs) (A) cells alone, (B) oxidized dextran (Odex)/collagen (Col), and (C) Odex/Col with laminin (LMN); (D, E) proliferation of HUVECs on the scaffolds without or with LMN. Asterisk and bracket show significant differences within groups and dissimilar letters indicate a significant difference between groups (*p* < 0.05). A: Col (12 mg/mL); B: Col (6 mg/mL); C: Odex (30 mg/mL)/Col (6 mg/mL); D: Odex (30 mg/mL)/Col (3 mg/mL); E: Odex (40 mg/mL)/Col (4 mg/mL); F: Odex (40 mg/mL)/Col (2 mg/mL); G: Odex (45 mg/mL)/Col (3 mg/mL); H: Odex (45 mg/mL)/Col (1.5 mg/mL).

Viability and proliferation of the HUVECs in different 3D scaffolds were determined by Live–Dead and Alamar blue assay (Figure 2A–E). The qualitative assessment of the HUVECs using Live–Dead staining revealed that adding LMN to the scaffold improved HUVEC growth. No detectable dead cells were found in the fluorescence microscopy images. Besides, the cell density enhanced significantly in the group “A” as determined by the Alamar blue assay after 72 h. As the Col concentration was reduced (group “B”), cell viability decreased by about 30% relative to the initial cell seeding density. In contrast, Groups “D,” “F,” and “H” prepared either at 25 or 37°C showed significantly higher cell viability. Groups “C” and “E” also had enhanced cellular viability, although it was not statistically significant. When cell growth in groups “E” and “F” was compared, group “E” with a relatively higher amount of Col had significantly superior cell growth. Also, there was no statistically significant difference in cell viability between hydrogel scaffolds made at 25 and 37°C. Cell viability was re‐checked in combination with LMN after choosing groups “A,” “C,” and “E” based on cell viability, SEM, and rheological parameters. In general, LMN addition enhanced HUVEC growth compared to LMN‐free scaffolds, although the changes were not statistically significant. Importantly, viability increased considerably in the group “A” compared to Matrigel at both preparation temperatures and with or without LMN (*p* < 0.05) (Figure 2D,E).

**FIGURE 3:**
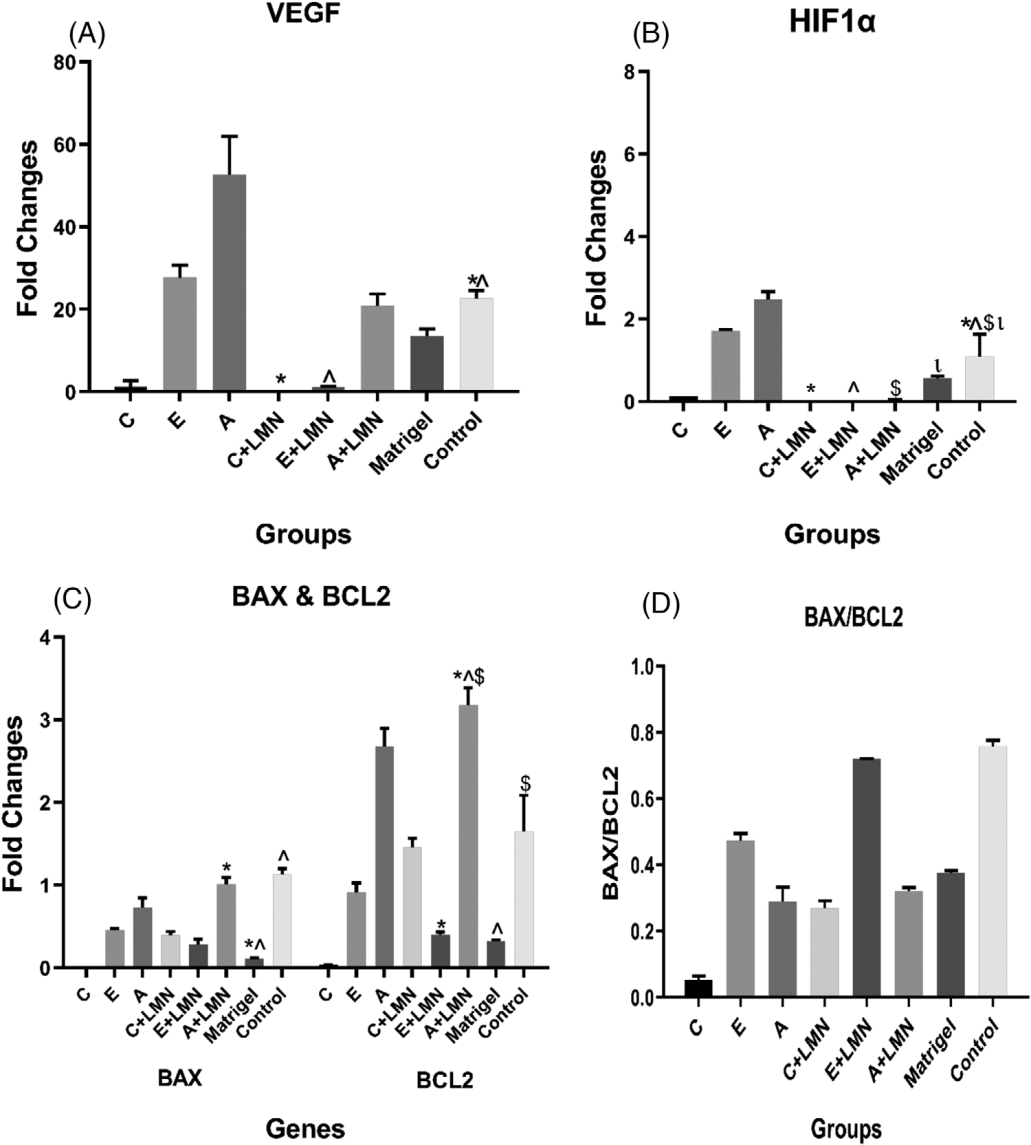
Gene expression in human umbilical vein endothelial cells (HUVECs) after culture in three‐dimensional (3D) scaffolds. Groups with the same superscripts were significantly different at *α* = 0.05 (*p* < 0.05). *Bax*, Bcl‐2‐associated X protein; *Bcl‐2*, B‐cell lymphoma 2; Col, collagen; *HIF1ɑ*, hypoxia inducible factor 1 subunit alpha; LMN, laminin; Odex, oxidized dextran; *VEGF*, vascular endothelial growth factor. A: Col (12 mg/mL); B: Col (6 mg/mL); C: Odex (30 mg/mL)/Col (6 mg/mL); D: Odex (30 mg/mL)/Col (3 mg/mL); E: Odex (40 mg/mL)/Col (4 mg/mL); F: Odex (40 mg/mL)/Col (2 mg/mL); G: Odex (45 mg/mL)/Col (3 mg/mL); H: Odex (45 mg/mL)/Col (1.5 mg/mL).

Real‐time PCR was used to assess HUVEC function biomarkers. As shown in Figure 3A, the highest level of *VEGF‐A* expression was found in group “A” without LMN. The results revealed a significantly lower *VEGF‐A* expression in group “C” and “E” with LMN compared to the control group (*p* < 0.05). Nevertheless, the differences between Matrigel and the other groups were not determined to be statistically significant. The results also showed no significant difference between the groups without LMN and the control group regarding the *HIF1α* expression. However, the *HIF1α* expression was downregulated in the groups containing LMN, which was significantly different from the control group (*p* < 0.05). Moreover, the level of *HIF1α* expression was higher in groups “A” and “E” without LMN compared to the other groups (Figure 3B). Importantly, the analysis of apoptotic (BAX) and anti‐apoptotic (BCL2) gene expression, as well as the BAX/BCL2 ratio (known as the apoptosis index), revealed that all treated groups had a lower apoptosis index than the control group. This decrease was more prominent in group “C” without LMN but did not differ substantially from the control group (Figure 3C,D).

**FIGURE 4:**
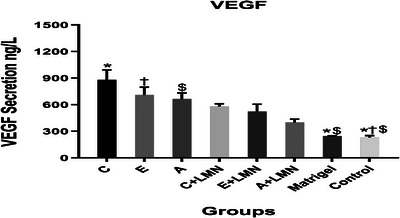
VEGF protein secretion. Groups with the same superscripts were significantly different at *α* = 0.05 (*p* < 0.05). Col, collagen; LMN, laminin; Odex, oxidized dextran; VEGF, vascular endothelial growth factor. A: Col (12 mg/mL); B: Col (6 mg/mL); C: Odex (30 mg/mL)/Col (6 mg/mL); D: Odex (30 mg/mL)/Col (3 mg/mL); E: Odex (40 mg/mL)/Col (4 mg/mL); F: Odex (40 mg/mL)/Col (2 mg/mL); G: Odex (45 mg/mL)/Col (3 mg/mL); H: Odex (45 mg/mL)/Col (1.5 mg/mL).

VEGF protein secretion was determined by the ELISA technique. Interestingly, the VEGF protein secretion increased in various groups treated either with or without LMN compared to the Matrigel and control groups, with a significant difference between the groups treated without LMN and the control group (*p* < 0.05). There was also a significant difference between groups “A” and “C” and the Matrigel group (*p* < 0.05). Moreover, the groups without LMN produced higher levels of the VEGF protein compared to those with LMN (Figure 4).

## 4 DISCUSSION

A favorable scaffold should be biocompatible and biodegradable, as well as mimic the mechanical and structural properties of ECMs. Many ECM components have proangiogenic properties and may promote endothelial cell survival, growth, migration, and/or tube formation, including Col, LMN, and fibronectin. Over the past two decades, many attempts have been made to introduce new tissue substitutes to promote angiogenesis [2, 3, 40–42]. Angiogenesis is a fundamental step in TE for oxygen and nutrients transport, and new blood vessels must be formed quickly and effectively for a successful transplantation [1]. In this context, scaffolds, particularly those based on Col, have been utilized. Nevertheless, their application has been limited because of their poor mechanical strength. One approach to overcoming this limitation is to combine scaffolds with another material that can act as a cross‐linker. Odex is a biocompatible hydrophilic cross‐linker that has been used in a wide range of applications for cell culture and controlled drug release [43, 44]. According to few studies [45, 46, 47], Odex can be employed as a cross‐linker to improve mechanical and chemical properties. Dextran monomer has two sites that are cleaved by periodate oxidation (C2–C3 and C3–C4), breaking the structure of the glucopyranose ring and forming crosslinking aldehyde groups [21]. In the present study, after oxidizing dextran, different ratios of Col were combined with Odex to fabricate an array of scaffolds at 25 and 37°C to promote angiogenesis. According to the results of rheometry and SEM experiments, rapid development of porous scaffolds with high elastic modulus (*G*′) was achieved in the Odex (4 mg/mL)/Col (40 mg/mL) group (“E”) at 37°C. In addition, when Odex (concentration > 30 mg/mL) was combined with Col (concentration > 3 mg/mL) at 37°C, stiffer scaffolds were formed compared to pure Col (groups “A” and “B”). These findings showed that the optimal temperature for crosslinking Col with Odex was 37°C. In a study by Zhang et al., mixing Odex with Col improved the mechanical property of Col was improved. Accordingly, *G*′ of Odex/Col (1:1) and Col at 37°C was about 5 and 100 Pa, respectively [27]. In another study, the *G*′ was increased from 0.1 to 1.0 kPa by crosslinking Col with genipin (10 mmol/L) [48]. Moreover, large pores were observed at high Odex concentrations. The scaffolds prepared at the lower temperature (25°C) also had larger fiber diameters and pore sizes. By increasing the temperature (37°C), the hydrogels displayed a fine and uniform nanofibrous structure. Overall, Odex/Col hydrogel scaffolds surpassed Col alone due to their reproducible structure, tunable porosity, mechanical properties, and biochemical functionalities [49, 50].

Alamar blue assay can be used to determine the intracellular metabolic activity. Resorufin, which was produced enzymatically in this research, is water soluble and non‐toxic. Thus, it can be measured directly in culture constructs. This process is accompanied by a change in color from indigo blue to pink. The present study confirmed that Odex had no cytotoxic effects on cells and could promote cell proliferation. Similarly, Zhang et al. reported that Odex/Col scaffolds had no cytotoxicity on L929 fibroblast cells after 72 h [27]. Another study conducted by Balakrishnan et al. demonstrated that Odex in combination with chitosan was compatible with fibroblasts, with the cells retaining their normal spindle shape [51]. The current study results revealed a higher cell proliferation in the Col scaffold at 37°C than in the other groups. Additionally, HUVECs grew better in the Odex/Col scaffold prepared at 37°C compared to 25°C. This might be due to improved crosslinking at 37°C compared to 25°C. Overall, Col provides both biological cues and structural support for cells through a dense fibrillar network [52]. Nonetheless, it has been demonstrated that Col‐based materials degrade over time and contract in the cell culture [53, 54], and crosslinking (e.g., with Odex) can overcome this issue. After evaluating the characteristics of Odex/Col scaffolds, LMN was chosen as an ECM component to improve the migration and proliferation of endothelial cells in the hydrogel scaffolds. LMN plays a fundamental role in angiogenesis by directly affecting gene and protein expression profiles in endothelial cells [55]. Compared to the groups without LMN, LMN increased the viability of the cells grown onto the scaffolds, but the difference was not significant. Similarly, DeHahn et al. emphasized that LMN supported endothelial cell survival [56].

To explore the biological behavior of the HUVECs grown onto the fabricated scaffolds, the expression levels of the most important genes and VEGF secretion were evaluated. The results revealed that the expression levels of the *VEGF* gene and its protein were higher in the groups without LMN. It has been suggested that due to the expression of the LMN receptor in HUVECs, LMN could inhibit the Toll‐like receptor (TLR) signaling pathway and subsequently decrease the expression of *VEGF* and its receptor [57, 58]. The expression level of the *VEGF* gene increased along with the expression of the *HIF1α* gene in all study groups, and the expression of both genes reduced by adding LMN, which may be linked to the TLR/HIF/VEGF signaling pathway and the detrimental effect of LMN on TLR [57, 58, 59, 60]. Apart from *VEGF* and *HIF1α* expression, the apoptosis index is another key determinant of the HUVEC viability and function. The Odex (30 mg/mL)/Col (6 mg/mL) group had the lowest apoptosis index (BAX/BCL2 ratio), indicating that the HUVECs were able to survive and function (VEFG protein secretion) effectively. The order of VEGF protein secretion in the groups did not correspond well with VEGF gene expression, indicating that scaffold has an influence not only on expression but also on VEGF uptake by HUVECs [31]. Overall, HUVECs proliferated and survived more successfully in the scaffold with high amount of Col with or without LMN addition.

## 5 CONCLUSION

Odex was synthesized and used as a natural polysaccharide derivative to crosslink Col and prepare hydrogel scaffolds for TE. The study was also aimed to incorporate LMN as an angiogenic ECM component for promoting HUVEC cell proliferation and function. Odex/Col hydrogels were rapidly formed at a relatively low Odex and high Col concentrations. SEM observations revealed that the Odex/Col hydrogels had a more regular 3D structure with large pores when compared to the pristine Col hydrogel. Pore size, gelation time, and hydrogel stiffness may all be modified by varying Col concentration, Odex/Col ratio, and temperature to meet the requirements for cell growth and survival. Our results showed that the Odex/Col scaffold had a reduced cell apoptosis index whereas the Col scaffold promoted HUVEC growth. Besides, LMN addition did not essentially result in improved HUVEC function. Overall, the results indicated that Odex/Col could be a viable option for TE, however more in vitro and in vivo research is required to confirm angiogenesis.

## CONFLICT OF INTEREST STATEMENT

The authors declare that they have no conflict of interests.

## Supporting information

Supporting Information

Supporting Information

## Data Availability

The datasets generated during and/or analyzed during the current study are not publicly available due to our center policy but are available from the corresponding author on request.
