# Supplementary material for: Biocompatible scaffolds based on collagen and oxidized dextran for endothelial cell survival and function in tissue engineering
Source: Eng Life Sci. 2023 Jun 13;23(7):2200140. doi: 10.1002/elsc.202200140 (PMC10317976; doi:10.1002/elsc.202200140)
Supplement: Supplementary file 1 — Supporting Information [file ELSC-23-2200140-s002.docx]

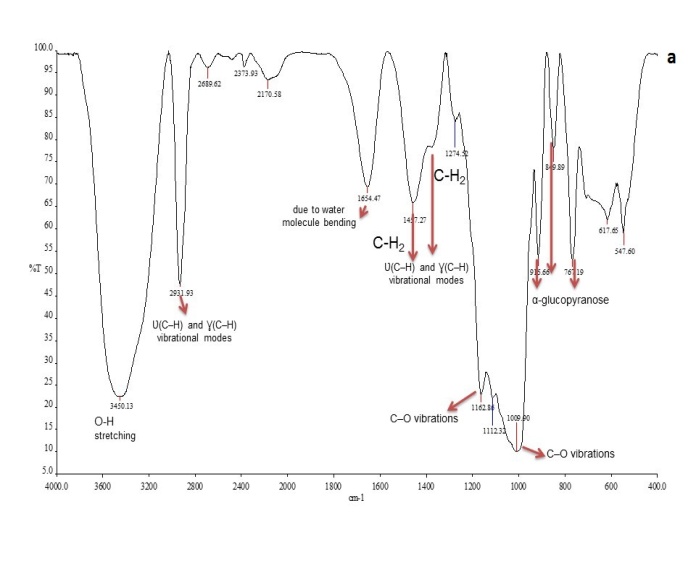

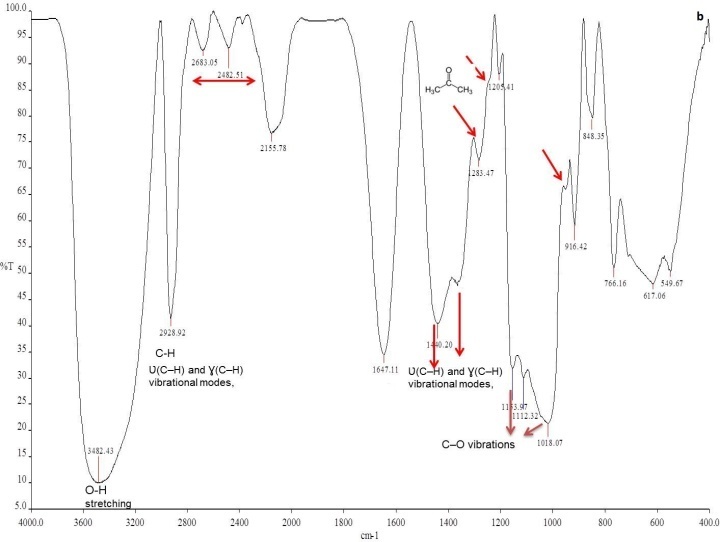


**Figure 1.** FTIR spectra of dextran (a) and Oxidized Dextran (Odex) (b). The inset arrows represent the spectra at the specified range, exhibiting differences in the peaks of both spectra.


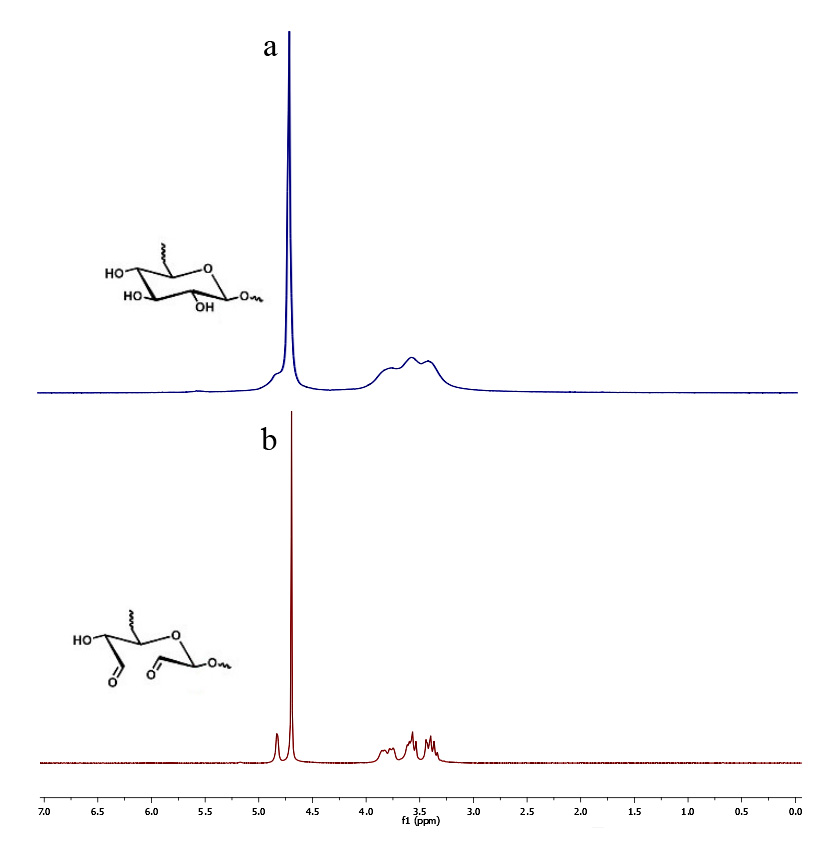


**Figure 2.**^1^H-NMR spectra. a. Dextran, b. Oxidized Dextran.
