# Supplementary material for: Biocompatible scaffolds based on collagen and oxidized dextran for endothelial cell survival and function in tissue engineering
Source: Eng Life Sci. 2023 Jun 13;23(7):2200140. doi: 10.1002/elsc.202200140 (PMC10317976; doi:10.1002/elsc.202200140)
Supplement: Supplementary file 2 — Supporting Information [file ELSC-23-2200140-s001.docx]

**Reological graph**
